# Optical meta-atom for localization of light with quantized energy

**DOI:** 10.1038/ncomms9766

**Published:** 2015-10-30

**Authors:** Sylvain Lannebère, Mário G. Silveirinha

**Affiliations:** 1 Department of Electrical Engineering—Instituto de Telecomunicações, University of Coimbra, Coimbra 3030-290, Portugal

## Abstract

The capacity to confine light into a small region of space is of paramount importance in many areas of modern science. Here we suggest a mechanism to store a quantized ‘bit' of light—with a very precise amount of energy—in an open core-shell plasmonic structure (‘meta-atom') with a nonlinear optical response. Notwithstanding the trapped light state is embedded in the radiation continuum, its lifetime is not limited by the radiation loss. Interestingly, it is shown that the interplay between the nonlinear response and volume plasmons enables breaking fundamental reciprocity restrictions, and coupling very efficiently an external light source to the meta-atom. The collision of an incident optical pulse with the meta-atom may be used to release the trapped light ‘bit'.

## Abstract (web summary)

Plasmonics enables the medication of light on length-scales smaller than the wavelength of the light. Here, the authors present a theoretical investigation of optical meta-atoms based on core-shell plasmonic nanostructures, where light is confined into a small region of space.

## Graphical abstract

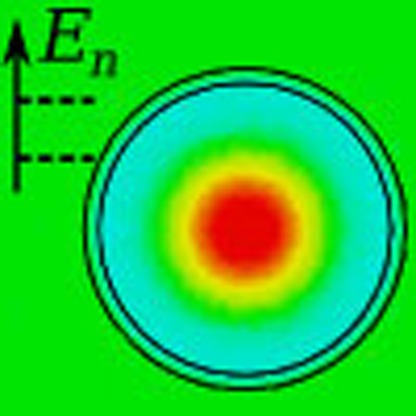

Light localization has several important technological applications^1^, and is usually achieved with the help of physical barriers such as mirrors^2^ and photonic band-gap materials^3^,^4^ that act to strongly reduce the effects of radiation loss. Light is however an object difficult to tame: no matter how elaborate and intricate are the material constructions that may be used to screen it from the exterior environment there is always some residual coupling with the radiation continuum, and hence light—if not absorbed by the material walls—always finds its way out. Indeed, in any conventional open resonator (for example, whispering gallery resonators^5^,^6^, or metallic nanoparticles^7^,^8^) the coupling to the surrounding region is never totally suppressed, and radiation loss is one of the factors that limits the lifetime of light oscillations. Even though the radiation emission due to specific electric current oscillation modes may be residual (for example, the anapole current distribution^9^), it is never precisely zero.

In the last decades, there has been a great interest in alternative mechanisms to localize light within the radiation continuum^10^,^11^,^12^,^13^,^14^,^15^,^16^,^17^. An interesting approach is based on an old idea by von Neumann and Wigner who discovered that certain electric potentials may support spatially localized electron states with energies higher than the potential barriers^18^,^19^. In recent years, different groups have extended this idea to light waves, and it has been shown that open material structures with tailored geometries may support localized light states^11^,^12^,^13^,^14^,^16^,^17^. In these structures, the discrete light spectrum overlaps the continuous spectrum, and hence these spatially localized states with infinite lifetimes are technically known as ‘embedded eigenvalues'. The existence of such states is highly nontrivial and truly remarkable, because it shows that light may be localized based simply on the scattering provided by a set of surrounding transparent (ideally lossless) material objects. There is however a downside: until recently, the known solutions were based on infinitely extended material profiles, for example, a photonic crystal. Thus, the objects that localize the radiation are required to be placed also at arbitrarily large distances from the spot wherein the light is concentrated. If the structure is truncated the localization becomes imperfect, and the oscillation lifetime becomes finite.

Recently, we introduced a novel approach to trap light in a bounded open cavity with suppressed radiation loss^20^. It was theoretically shown that under some strict conditions, volume plasmons—that is, charge density waves in metals—may enable the formation of ‘embedded eigenvalue' states in finite sized cavities, such that in the limit of no material loss the light oscillations can have infinitely long lifetimes. Our proposal applies to a wide range of resonators, and in particular the light volume may be ultra-subwavelength^15^,^20^. In a two-layer spherical structure, the shell is ideally formed by a material with vanishing permittivity 
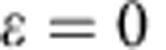
—so that it supports volume plasmons—and the core region is a vacuum or a standard dielectric^20^.

A limitation of this system is that because of the Lorentz reciprocity theorem the trapped light state cannot be pumped by an external source. Indeed, structures made of reciprocal materials are intrinsically bi-directional, and hence if the trapped light cannot leak out then it is also impossible to feed its oscillations with an external excitation^20^. Here it is shown that the interplay of volume plasmons with a nonlinear material response may provide the means to pump the embedded eigenstate using an external source. Notably, it is proven that the energy associated with the embedded eigenvalue is quantized, such that self-sustained oscillations are only possible for specific stored energy values. Because of the obvious parallelisms with the energy quantization of bound electronic states in atoms, we refer to the proposed resonator as an ‘optical meta-atom'. In realistic systems, the material absorption limits the number of cycles during which the radiation can be trapped in the optical meta-atom. In principle, this limitation may be compensated using a gain medium. It is envisioned that a gain compensated meta-atom may be used as an elementary one-bit optical memory.

## Results

### The meta-atom

**Figure 1:**
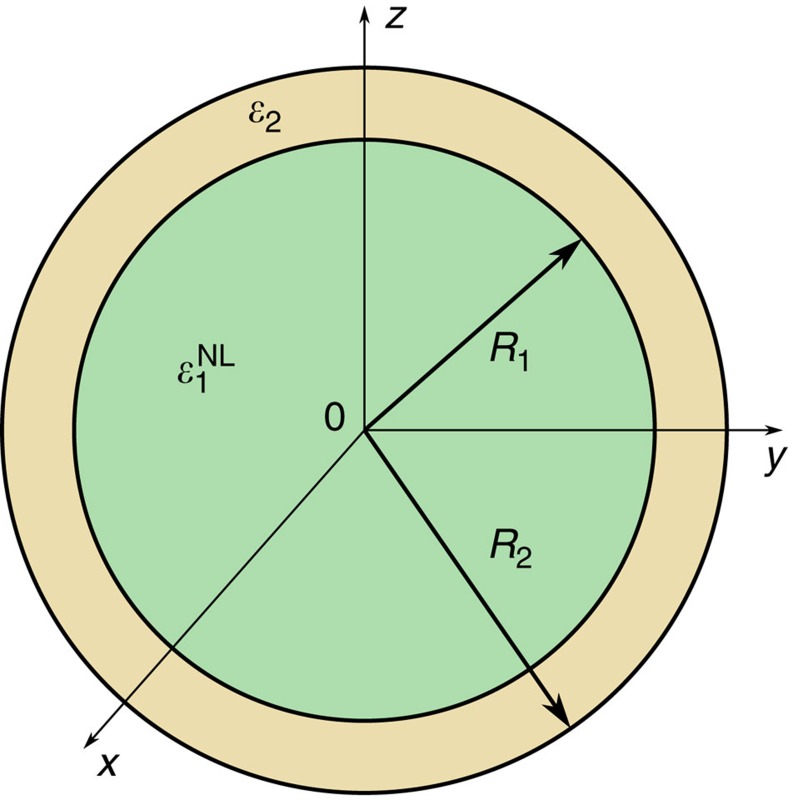
The optical meta-atom. The core material is a dielectric with a nonlinear permittivity response 
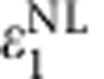
, while the shell is a plasmonic material with permittivity 
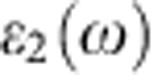
. The inner and outer radii are *R*_1_ and *R*_2_, respectively.

The geometry of the core-shell spherical optical meta-atom is represented in Fig. 1. The shell has radius *R*_2_, and, without loss of generality, its relative permittivity is supposed to follow a Drude dispersion model 
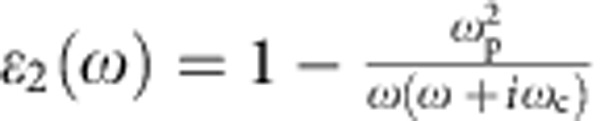
, where *ω*_p_ is the plasma frequency and *ω*_c_ is the collision frequency. The core has radius *R*_1_ and is made of a dielectric material with relative permittivity 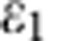. As shown in our previous work, the volume plasmons in the shell at *ω*=*ω*_p_ may perfectly screen the light in the core region, so that in the limit of vanishing material loss (*ω*_c_→0) the oscillations lifetime may be infinite^20^. This effect requires that at *ω*=*ω*_p_ one has *j*_*n*_(*k*_1_*R*_1_)=0, where *j*_*n*_ is the spherical Bessel function, 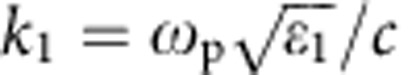, and *n*≥1 is the azimuthal quantum number. For a fixed 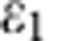, this condition is satisfied only for certain specific values of the core radius. For instance, for *n*=1 the trapped fields have a dipolar (*p*-type) symmetry and the first zero of *j*_1_(*u*) occurs at *u*≈4.49, so that the corresponding optimal radius is 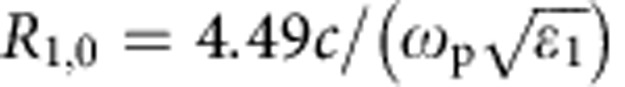
(ref. 20). Interestingly, in the ideal regime 
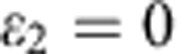
 the radiation is held within the core forever, even for a deeply subwavelength shell. This contrasts with conventional high-Q optical cavities. For instance, we numerically verified (not shown) that to achieve a quality factor of the order of 100 in a Fabry–Perot cavity with lossless photonic crystal walls, the mirror walls need to be at least six wavelengths thick. Importantly, any perturbation of the optimal radius, no matter how small, will lead to a finite oscillation lifetime^20^. Moreover, as discussed previously, even if the inner radius could be tuned to exactly satisfy *R*_1_=*R*_1,0_, it would still be impossible to externally pump the light oscillations.

To overcome these restrictions, here we investigate the opportunities created by a core region with an optical nonlinearity. It is assumed that the core material has an instantaneous isotropic Kerr response described by the nonlinear third-order susceptibility *χ*^(3)^ (ref. 21), such that the electric displacement vector satisfies 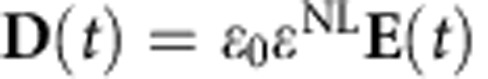, with 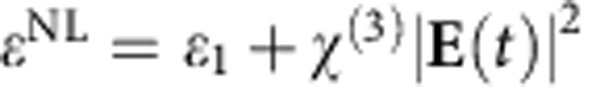 where 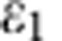 is the relative dielectric permittivity for weak field intensities. The key idea is to choose an inner radius *R*_1_ slightly different from *R*_1,0_ (so that the field oscillations can be externally pumped), and take advantage of the nonlinear dynamics to self-tune the resonator. Heuristically, one may expect that when 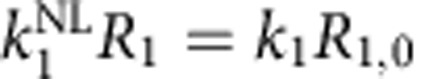 the radiation loss may be strongly suppressed, where we put 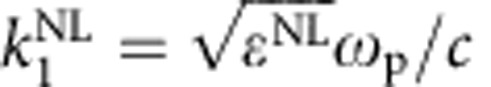. This condition is equivalent to:


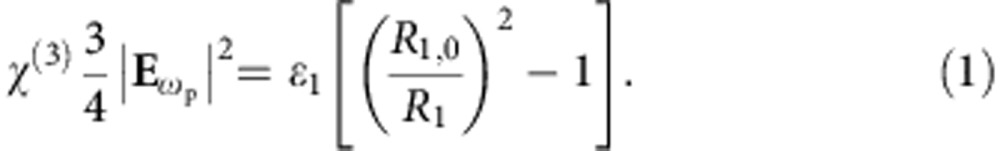


Note that if 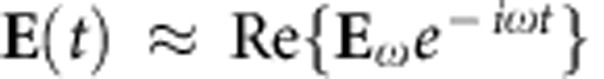 then 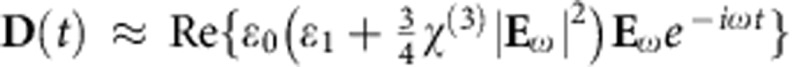 for a weak nonlinearity. This result was used to estimate 
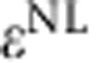
 in the core region. Hence, for a self-focusing Kerr material (*χ*^(3)^>0) the resonator self-tuning may be feasible when *R*_1_<*R*_1,0_.

### Temporal dynamics of the electromagnetic field

To put these ideas on a firm ground, next we develop a simple analytical model for the nonlinear dynamics of the core electric field in a scenario wherein the optical meta-atom is illuminated by an incoming plane wave. To begin with, we consider the linear case (*χ*^(3)^=0) and note that under plane wave incidence, the electric field at the centre of the core-shell particle is given in the frequency domain by 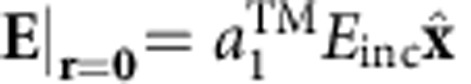 (refs 20, 22). Here, 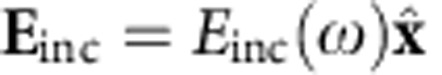 is the linearly polarized incident field calculated at the centre of the particle and 
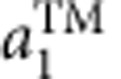
 is the first order Mie coefficient for transverse radial magnetic (TM^*r*^) waves. It was shown in ref. 20 that for *R*_1_≈*R*_1,0_ and *ω*≈*ω*_p_ the Mie coefficient 
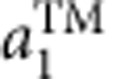
 satisfies:


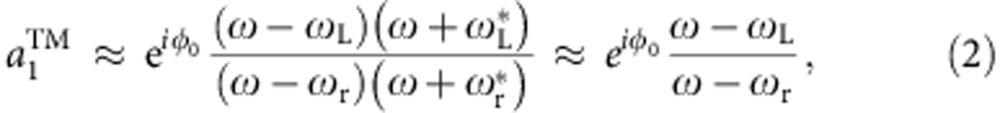


where *φ*_0_(*ω*) is some phase factor associated with a time delay, and the second identity assumes that both *ω*_L_ and *ω*_r_ are near *ω*_p_. In the above, 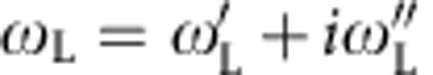 is the complex frequency associated with plasmon oscillations, which is defined by 
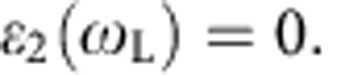
 Moreover, 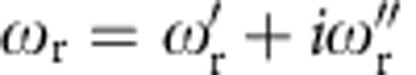 is the complex resonance frequency of the trapped mode, and it may be numerically calculated as explained in ref. 20. When 
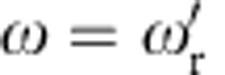
 the inner field may be strongly enhanced due to the excitation of a trapped state with quality factor 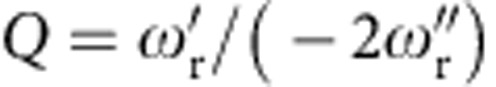, whereas when 
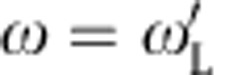
 it is near zero due to the screening provided by the volume plasmons. The response has a Fano-type lineshape^8^.

If the spectrum of the incident field is concentrated at *ω*_p_, it is possible to write in the time domain 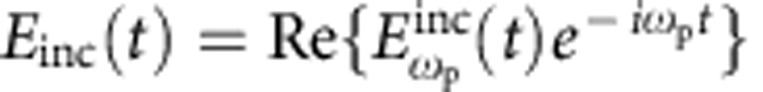 and 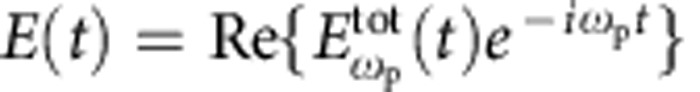, with the envelopes of the incident field 
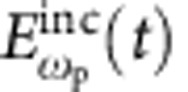
 and of the total field 
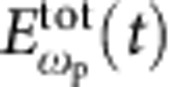
 varying slowly in time. Calculating the inverse Fourier transform of 
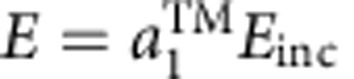
, it is readily found that the differential equation governing the time evolution of the total field envelope at the centre of the core-shell particle is:


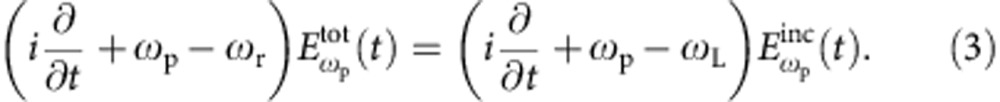


For simplicity the phase factor *φ*_0_ was dropped.

**Figure 2:**
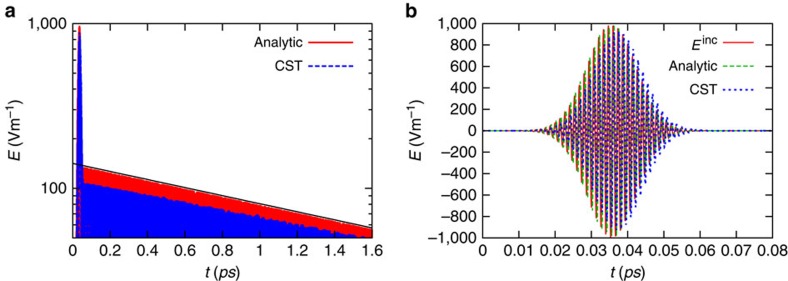
Excitation of the meta-atom in the linear regime. Electric field at the centre of the meta-atom as a function of time. The incident pulse duration is 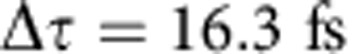. (**a**) *R*_1_≈0.98*R*_1,0_. The black thin line represents the theoretical peak amplitude determined by the decay rate 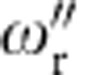. (**b**) *R*_1_=*R*_1,0_.

To validate this theory, next it is assumed that the inner core has a linear response with permittivity 
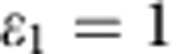
 and that the plasmonic shell has radius *R*_2_=1.1*R*_1_ described by a Drude model with the plasma frequency *ω*_p_/2*π*=750 THz (violet light) and negligible material loss. For this configuration the optimal radius is *R*_1,0_≈286 nm. The core-shell particle is excited by an *x*-polarized incident field with a Gaussian profile in the time domain with envelope 
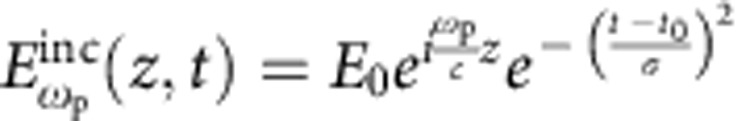
, propagating along the *z* direction. The time duration of the pulse is determined by the full-width-half-maximum 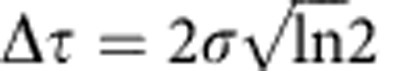, and *t*_0_ is the time instant for which the incoming wave field is peaked. First, we consider that *R*_1_≈0.98*R*_1,0_, and suppose that the incident pulse has a peak amplitude *E*_0_=1,000 Vm^−1^, and a duration 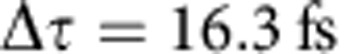. Figure 2a represents in a semi-logarithmic scale the *x* component of the electric field at the centre of the meta-atom as a function of time. The solid red line was calculated using the analytical model (3) and the dashed blue line was obtained with CST Microwave Studio^TM^ (ref. 23). As seen, apart from a small amplitude shift, the analytic and full-wave results are rather similar. This confirms that the differential equation (3) may be used to characterize the dynamics of the electric field in the core region. Remarkably, even though the sphere's diameter is comparable with the wavelength, the temporal evolution of the field inside the meta-atom is well described by the electric dipolar mode. Indeed, the meta-atom is designed to exclusively trap this oscillation mode and therefore the other modes, even if present, decay quickly after the initial excitation period. In the example under study, due to the relatively small size of the meta-atom the secondary resonance nearer *ω*_p_ occurs at *ω*≈0.8*ω*_p_ and has a quality factor ten times smaller than that associated with the primary resonance. This low quality factor is justified by the fact that the shell becomes weakly reflecting when the oscillation frequency drops to 0.8*ω*_p_.

Notably, after the incident pulse overtakes the meta-atom, the field continues to oscillate inside the core with a peak oscillation amplitude that decays exponentially. The peak amplitude is well described by the formula 
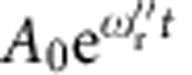
 (black line in Fig. 2a), where *A*_0_ is some fitting constant and 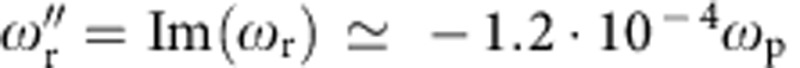 is the decay rate calculated with the theory of ref. 20. Note that the free oscillation amplitude is significantly less than *E*_0_ and, very importantly, it is strictly linked to the free oscillation decay rate. To show this, we plot in Fig. 2b the electric field at the centre of a meta-atom with *R*_1_=*R*_1,0_. The incident field is the same as in the previous example. Crucially, the field inside the meta-atom vanishes almost instantaneously after the end of the incident pulse, notwithstanding that for this configuration the theoretical decay rate vanishes: 
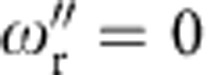
. This result confirms that for a perfectly tuned cavity it is impossible to pump the ‘embedded eigenvalue' state with an external excitation, in agreement with the Lorentz reciprocity theorem^20^. Indeed, in the linear lossless regime, increasing the trapped state lifetime (that is, decreasing the scattered power) inevitably implies decreasing the external coupling strength (that is, the extracted power; see 24,25).

### Trapping a light bit

To show how one may take advantage of the nonlinear dynamics to surpass these fundamental limitations, next equation (3) is generalized to include the effect of a nonlinear material response. To do this, *ω*_r_ is regarded as a function of the inner core permittivity 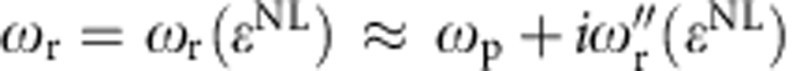. The decay rate 
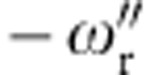
 has a minimum 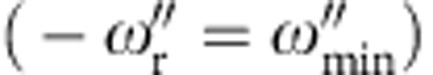 for the optimal inner core permittivity 
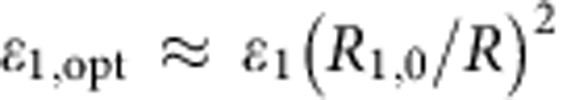
. The minimum is proportional to the decay rate of the volume plasmon oscillations: 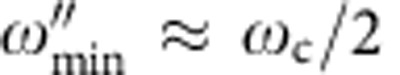 (ref. 20). Thus, one may write:


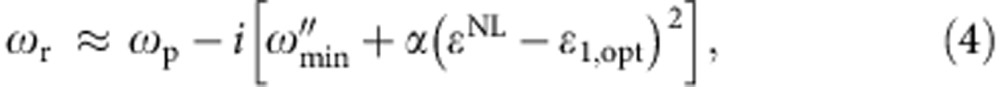


where 
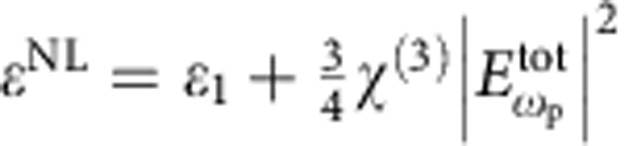
, and α is some constant that can be obtained from a Taylor expansion of the formula 
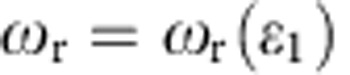
 around 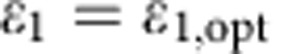. For example, for *R*_2_=1.1*R*_1_, and *R*_1_=0.98*R*_1,0_ it is found that *α*≈0.072*ω*_p_. Substituting equation (4) into equation (3) it is possible to characterize the nonlinear dynamics of the system. Importantly, for time intervals wherein the incident field vanishes (for example, after the incoming pulse overtakes the meta-atom) the inner field envelope is determined by 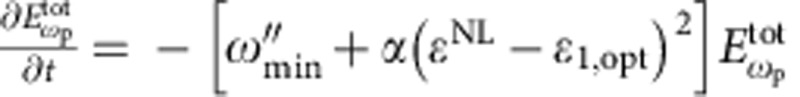. Hence, if the plasmons decay rate is negligible or if it is compensated by some gain mechanism 
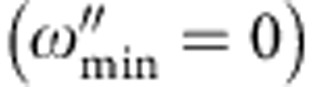
, it is possible to suppress the radiation loss when 
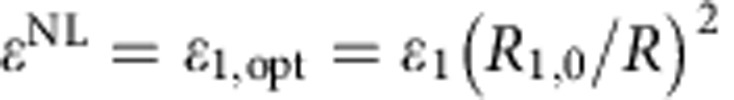
. This condition is equivalent to equation (1), and implies that the stored field peak amplitude is precisely determined by the strength of the nonlinearity. Thus, a nonlinear material response may enable us to efficiently pump the embedded ‘eigenstate' with an external source, such that the trapped radiation can be retained within the resonator for an extremely long time, only limited by the material loss in the meta-atom. Moreover, equation (1) suggests that the amount of energy that may be retained within the optical meta-atom should be independent of the excitation. Thus, the energy of a trapped light ‘bit' is precisely quantized, and in principle can only have a single nonzero value determined by the value of *χ*^(3)^. For *n*=1, the trapped state is associated with dipolar-type oscillations and hence it is triply degenerate in the linear regime^20^. Thus, the optical meta-atom may be a photonic analogue of an atomic system with degenerate energy levels.

**Figure 3:**
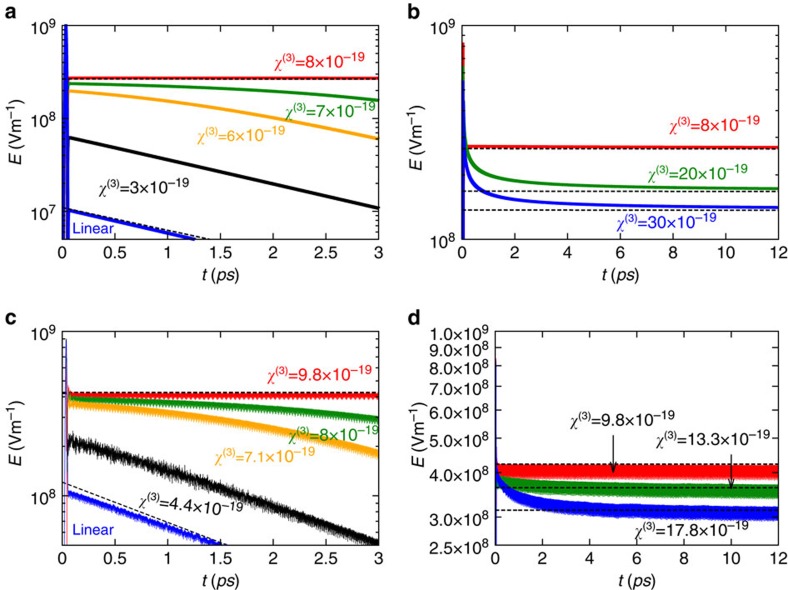
Excitation of the meta-atom in the nonlinear regime. Influence of the value of *χ*^(3)^ (in m^2^ V^−2^) on the trapped field decay for an incident pulse with 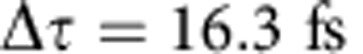. (**a**,**b**) Analytical model. (**c**,**d**) CST Microwave Studio simulations.

To illustrate the discussion, we show in Fig. 3 the dynamics of the peak electric field obtained by numerically solving (3) for different values of *χ*^(3)^. The structural parameters of the meta-atom are as in the previous examples (*R*_1_≈0.98*R*_1,0_ and *R*_2_=1.1*R*_1_), and the incident field amplitude is *E*_0_=10^9^ Vm^−1^. The incident pulse is peaked at *t*_0_=0.047 ps (sharp peak in Fig. 3) and its duration is determined by 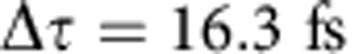. As seen in Fig. 3a, as *χ*^(3)^ is increased the external coupling efficiency is improved, whereas at the same time the decay rate approaches the minimum value 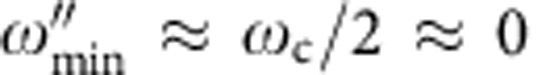. For *χ*^(3)^≥8·10^−19^ m^2^ V^−2^ (Fig. 3b), the field decay rate is 
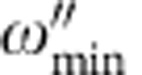
 and a light ‘bit' may stay trapped within the meta-atom for an extremely long period of time. Consistent with equation (1) this regime is characterized by a specific value of 
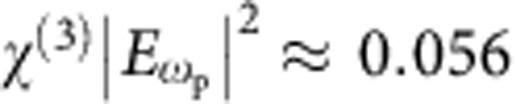
. It is important to highlight that (if different from zero) the trapped field steady-state amplitude only depends on the strength of the nonlinearity, and not on the excitation.

**Figure 4:**
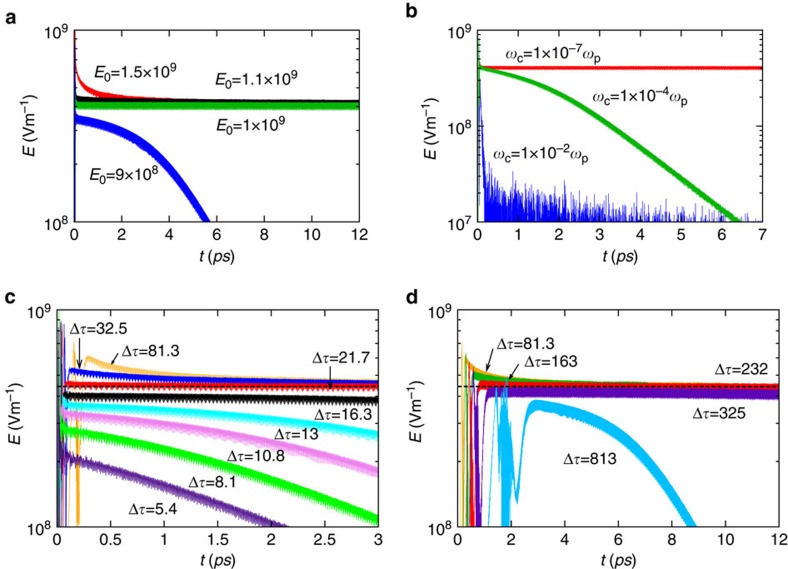
The effect of *E*_0_, material loss, and pulse duration on the field decay. Influence of (**a**) the incident field magnitude *E*_0_ and (**b**) the level of losses *ω*_c_ on the trapped field decay for a core material with *χ*^(3)^=9.8·10^−19^ m^2^ V^−2^ and an incident pulse of duration 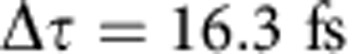. Influence of the pulse duration (in fs) on the trapped field decay for a core material with *χ*^(3)^=8.89·10^−19^ m^2^ V^−2^. (**c**) Short-pulse duration. (**d**) Long-pulse duration.

To validate our analytical model, we did a similar study using CST Microwave Studio (Fig. 3c,d). As seen, the full-wave CST simulations are qualitatively consistent with the results of the analytical model. Now, the radiation loss is suppressed for *χ*^(3)^≥9.8·10^−19^ m^2^ V^−2^. The threshold for light trapping is 
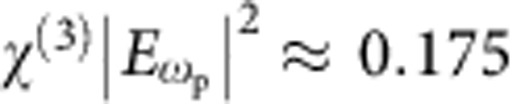
, which is about three times larger than the value predicted by the analytical model. The threshold value can be made arbitrarily small by reducing the detuning of *R*_1_ with respect to *R*_1,0_. Notably, for *χ*^(3)^=9.8·10^−19^ m^2^ V^−2^, the field amplitude at the centre of the meta-atom reaches, almost immediately after the end of the incident pulse, the steady-state value (indicated by the dashed horizontal lines in Fig. 3b) determined by the quantized value of 
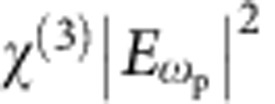
. The electric field time animation for the example of Fig. 3c with *χ*^(3)^=9.8·10^−19^ m^2^ V^−2^ can be found in the Supplementary Movie 1. For a third-order susceptibility greater than this value, the steady-state is only reached after the extra-amount of electromagnetic energy is released from the meta-atom. These results are confirmed by the study in Fig. 4a of the influence of the incident pulse amplitude for a fixed value of the third-order nonlinearity *χ*^(3)^=9.8·10^−19^ m^2^ V^−2^. As seen, for an amplitude above the threshold (*E*_0_=10^9^ Vm^−1^) and after relaxation of the extra energy, the field inside the core-shell particle always saturates at the same level.

As expected, the presence of material loss deteriorates the oscillation lifetime, but the results remain exciting for small values of the collision frequency. This is illustrated by Fig. 4b which represents the field at the centre of the meta-atom for different levels of loss in the plasmonic shell. Unfortunately, for a realistic level of metal loss it seems unfeasible to hold the light within the meta-atom for a large number of oscillation cycles. A solution that may help to alleviate this problem is to mimic the 
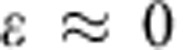
 regime using dielectric photonic crystals operating in the vicinity of a band-gap edge. In general, a practical realization of our idea may require some loss compensation mechanism^26^,^27^. Even though challenging, this may be within reach with the current state of the art technologies using either optical or electrical pumping^28^,^29^,^30^,^31^,^32^,^33^. Some encouraging results in this direction have been reported in the recent literature, specifically the realization of loss-compensated nanostructures relying on nanoscale gain media formed either by dye molecules (e.g., rhodamine dye) or by semiconductor nanostructures (e.g., quantum dots) such that the population inversion is created optically or electrically^26^,^27^,^34^.

The external coupling efficiency depends on the pulse duration 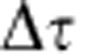. To illustrate this, we represent in Fig. 4c,d the inner field dynamics determined with CST Microwave Studio, for the case wherein the third-order susceptibility is kept constant *χ*^(3)^=8.89·10^−19^ m^2^ V^−2^ and the full-width-half-maximum of the incident pulse 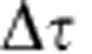 varies from 5.4 to 813 fs. As seen, for a pulse duration such that 5.4<
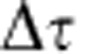
<21.7 fs the excitation efficiency is improved as 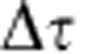 is increased, so that the field decay rate progressively reaches the optimal value 
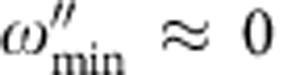
. For 21.7≤
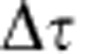
≤232 fs, the light can be trapped inside the particle with suppressed radiation loss. Again, for a pulse duration different from the threshold values (21.7 or 232 fs) the field does not reach immediately the quantized value of 
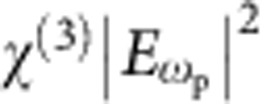
 and some extra time is needed to release the excess of electromagnetic energy. For 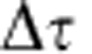
>232 fs, the external coupling efficiency is deteriorated and the temporal dynamics of the incident pulse is incompatible with the requirements for light trapping.

### Freeing the trapped light

In the limit of no material loss the oscillation lifetime may be extremely large (see Fig. 4b), possibly infinite. Parenthetically, we note that frequency conversion, namely third harmonic generation, may also limit the oscillations lifetime. Thus, from a theoretical point of view, it is interesting to discuss how a trapped light ‘bit' may be released from the meta-atom. We propose to do this using another Gaussian-shaped pulse. Hence, next it is supposed that the meta-atom is sequentially illuminated by two pulses. The first pulse has parameters *t*_0,1_=0.047 ps, duration 
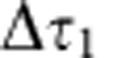
=21.7 fs and amplitude *E*_0,1_=10^9^ Vm^−1^, and serves to trap a light ‘bit' in the meta-atom. The core region is characterized by the susceptibility *χ*^(3)^=8.89·10^−19^ m^2^ V^−2^.

**Figure 5:**
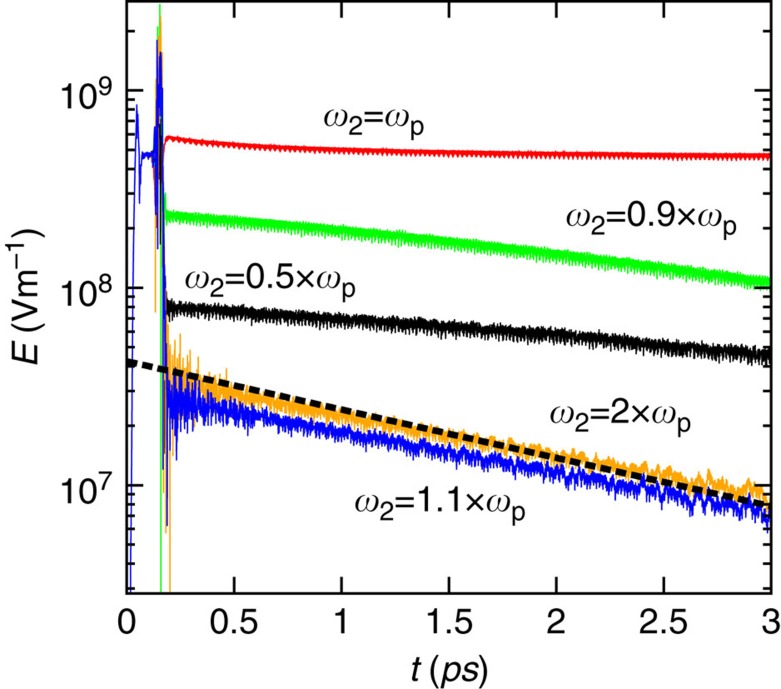
Release of the trapped light with a second light pulse. Electric field as a function of time when the meta-atom is sequentially illuminated by two-light pulses. The collision of the second pulse with the meta-atom (sharp peak in the plots) releases the trapped light ‘bit', except when the frequency of oscillation of the second pulse satisfies *ω*_2_=*ω*_p_. The dotted line represents the decay rate for the equivalent linear case.

One option to release the trapped light ‘bit' is to create a collision between a second light pulse with the same oscillation frequency *ω*_p_ and the meta-atom. We numerically verified that this is indeed a valid strategy (not shown), but in practice it may require very large field amplitudes (for example, 
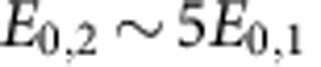
) and the impact of the second pulse with the meta-atom leads to a chaotic field behaviour. To circumvent this problem, we imagined a different solution relying on a light pulse with the same amplitude as the first one (*E*_0,2_=*E*_0,1_), but with a different oscillation frequency *ω*_2_. The remaining parameters of the second pulse are *t*_0,2_≈0.149 ps and 
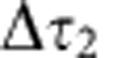
=28.3 fs. As seen in Fig. 5, the first light-pulse pumps the meta-atom ‘embedded eigenstate'. Notably, when the frequency of the second pulse *ω*_2_ is significantly different from *ω*_p_, its collision with the meta-atom frees the trapped light ‘bit'. This occurs because mixing the two pulses leads to both frequency and modal conversions. A time animation of the collision for the example of Fig. 5 with *ω*_2_=1.1*ω*_p_ can be found in the Supplementary Movie 2. Interestingly, when *ω*_2_>*ω*_p_ the light release occurs with a decay rate similar to the decay rate of the equivalent linear particle (with *χ*^(3)^=0). Moreover, the field relaxation after the second pulse overtakes the particle is faster for *ω*_2_>*ω*_p_. Note also that for *ω*_2_=*ω*_p_ the light ‘bit' remains trapped within the core after the collision with the second pulse.

## Discussion

In summary, we demonstrated that the interplay between a nonlinear response and plasmonic effects may enable storing a quantized amount of electromagnetic energy in an open plasmonic resonator. We investigated and characterized with full-wave simulations the conditions required for the meta-atom self-tuning. It was found that when the field amplitude and the pulse duration surpass certain thresholds, a well defined amount of energy stays trapped in the resonator. This regime with suppressed radiation loss is characterized by a specific value of 
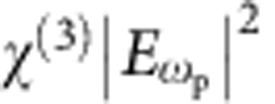
 close to the value predicted by equation (1). Hence, in a steady-state the trapped energy is independent of the excitation.

In practice, the lifetime of the trapped light bit is always limited by unavoidable material loss. One may envision that by incorporating some form of electrical or optical gain^28^,^29^,^30^,^31^,^32^,^33^ into the meta-atom it may be possible to compensate for the effects of absorption and hold the trapped light within the nano-resonator for a long period of time. The implementation of this gain compensation mechanism is at the present time the main challenge to render the light confinement in the optical meta-atom experimentally observable. In this regard, one should note that even if some gain element is incorporated into our proposal it remains fundamentally different from a nanolaser. Indeed, provided the level of gain in the linear regime is kept below the threshold associated with lasing (that is, the threshold required to overcompensate both the material absorption and the radiation loss) the system remains stable. The only way to trigger oscillations in the core region is by using an external optical excitation. Crucially, due to the nonlinear dynamics, the radiation loss of the system decreases when sufficient energy is pumped into the resonator. Hence, the gain required to compensate the total loss in the nonlinear regime is expected to be less than the threshold gain that drives the system into lasing in the linear regime. Because of this property, provided the gain response does not saturate in the dynamic range of interest, it may be feasible to guarantee a robust stable operation of the meta-atom in the linear regime and a full compensation of loss in the nonlinear regime. The combination of the meta-atom with a gain element may thus provide a rudimentary one-bit optical memory. It is relevant to mention that because the dimensions of the meta-atom are of the order of the wavelength its resonances are well separated in frequency. Thus, the presence of a gain medium with a spectral gain response peaked at the frequency *ω*_p_ is not expected to change in any manner the role of the secondary resonances, which have much lower quality factors.

Very importantly, our ideas may be generalized to other type of open resonators, and unveil a novel mechanism to break the Lorentz reciprocity principle and to efficiently couple high-Q optical resonators with an external excitation. In particular, our solution can be readily extended to more general geometries, not necessarily based on core-shell particles. Indeed, a generic high-Q optical resonator may be properly tuned such that when covered with an ideal 
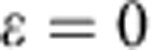
 shell it supports an embedded eigenvalue light state with suppressed radiation loss. Notably, these structures are expected to be much less sensitive to the effect of loss in the ENZ cover, because for a non-uniform core the task of confining the radiation within the meta-atom does not rely exclusively on the 
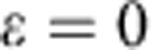
 cover. Thus, such modified meta-atoms may determine an exciting path towards the experimental verification of the light trapping with quantized energy.

Remarkably, a collision between another light pulse with different oscillation frequency and the meta-atom may enable releasing the trapped light ‘bit'. We envision that the developed ideas may find applications in chemical or biological sensing, light emitting sources and optical memories.

## Methods

The numerical results shown in Figs 2, 3, 4, 5 were obtained using the time domain solver of the commercial software CST Microwave Studio, using open boundary conditions and plane wave excitation. We numerically checked that our *χ*^(3)^ parameter is related to the definition adopted by CST as: 
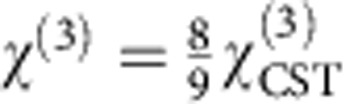
.

## Additional information

**How to cite this article:** Lannebère, S. & Silveirinha, G. M. Optical meta-atom for localization of light with quantized energy. *Nat. Commun.* 6:8766 doi: 10.1038/ncomms9766 (2015).

## Supplementary Material

Supplementary Movie 1 Time animation of the electric field corresponding to the example of Fig. 3c with *X*^(3)^ =9.8·10^−19^ m^2^ V^−2^

Supplementary Movie 2 Time animation of the electric field corresponding to the example of Fig. 5 with ω = 1.1ω_*p*_.
