# Supplementary figures and images for: Optical meta-atom for localization of light with quantized energy
Source: Nat Commun. 2015 Oct 30;6:8766. doi: 10.1038/ncomms9766 (PMC4640144; doi:10.1038/ncomms9766)

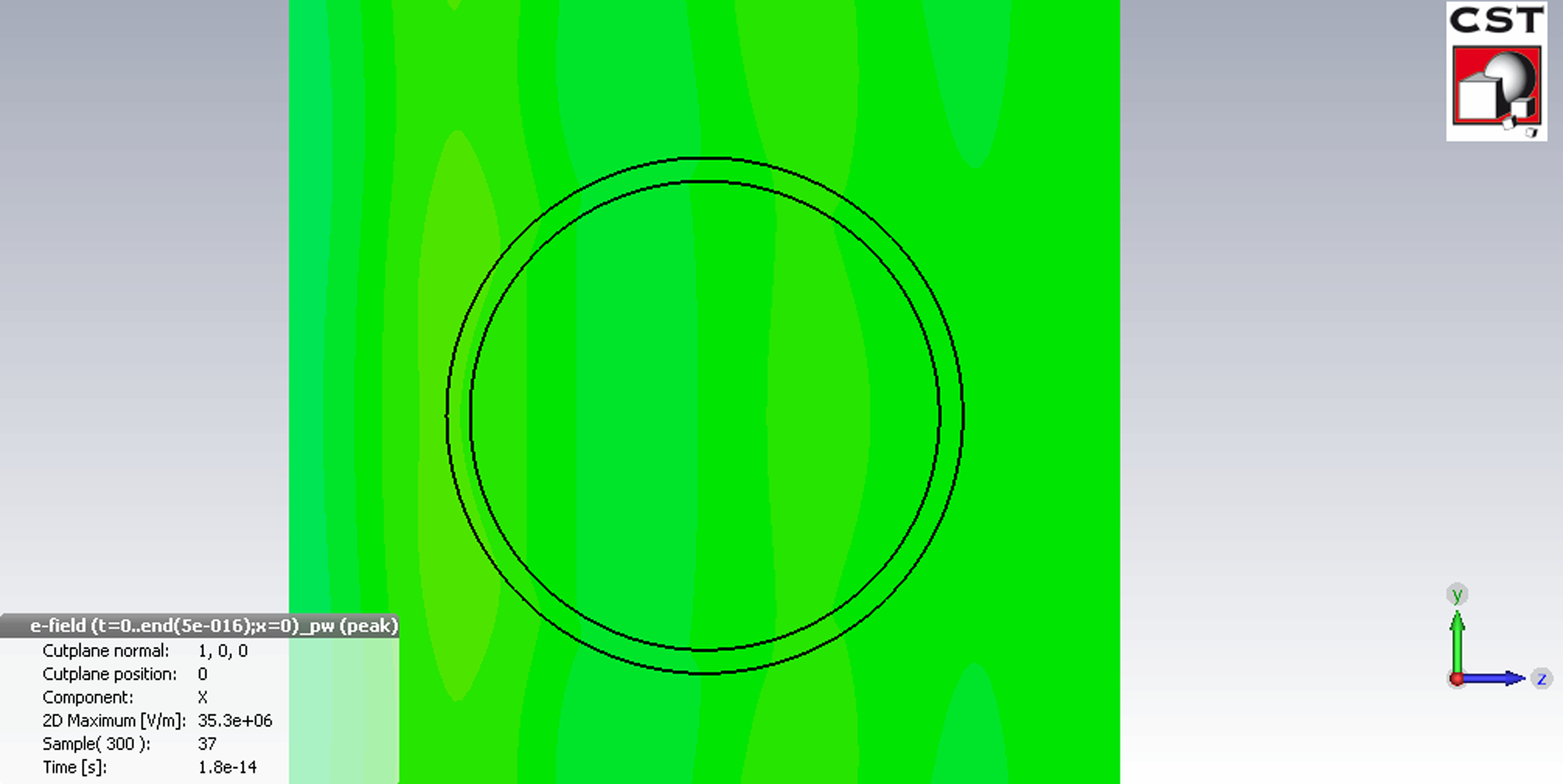

Supplement: Supplementary Movie 1 — Time animation of the electric field corresponding to the example of Fig. 3c with X(3) =9.8·10−19 m2 V−2 [file ncomms9766-s1.tif]

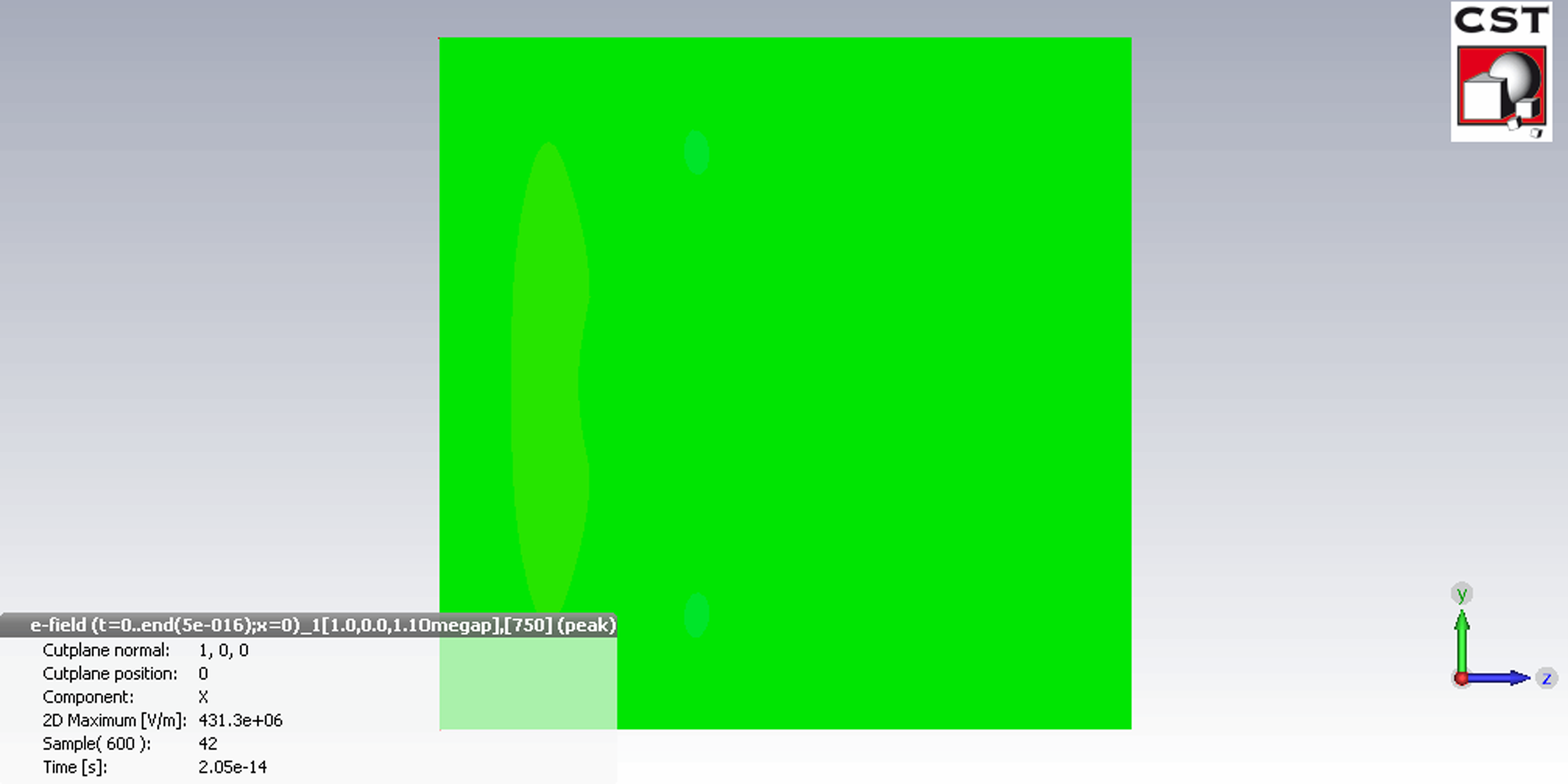

Supplement: Supplementary Movie 2 — Time animation of the electric field corresponding to the example of Fig. 5 with ω = 1.1ωp. [file ncomms9766-s2.tif]
